# Valorization of broccoli by-products: seasonal variations in bioactive compounds and their biostimulant effects on pak choi germination

**DOI:** 10.1371/journal.pone.0323848

**Published:** 2025-05-15

**Authors:** Lorena Albaladejo-Marico, Maria Gomez-Molina, Paula Garcia-Ibañez, Micaela Carvajal, Lucia Yepes-Molina

**Affiliations:** Aquaporins Group. Centro de Edafologia y Biologia Aplicada del Segura. C.E.B.A.S.-CSIC. Campus Universitario de Espinardo - 25, Murcia, Spain; Bangabandhu Sheikh Mujibur Rahman Agricultural University, BANGLADESH

## Abstract

The use of plant-based biostimulants is a sustainable strategy to enhance crop growth while mitigating the environmental impact of synthetic agrochemicals. Broccoli by-products, rich in bioactive compounds, have emerged as a promising resource, though their composition is influenced by plant growing conditions. This study investigates the biostimulant potential of broccoli-derived extracts obtained from leaves, stems, and petioles of plants cultivated in three different seasons (autumn, winter, and spring) and their effect on the germination and early growth of pak choi (*Brassica rapa* subs. *chinensis* L.) seeds. A comprehensive biochemical characterization, including mineral content, glucosinolates, and phenolic compounds, was conducted to explore how seasonal and tissue-specific variations impact their composition and biostimulant efficacy. Principal Component Analysis (PCA) revealed distinct metabolic profiles across seasons and plant tissues, with leaf-derived extracts showing higher correlations with phenolic acids and trace minerals, whereas petiole and stem extracts were associated with macronutrients. Germination assays demonstrated that extracts from autumn and winter exhibited the highest biostimulant activity, likely due to their enriched secondary metabolite profiles and well-balanced mineral composition. In contrast, spring extracts, despite their higher macronutrient content, showed limited biostimulant effects, possibly due to physiological constraints in broccoli plants during spring, when they experience reduced bioactive potential. PCA and correlation analysis identified metabolites, particularly sinapic acid and glucobrassicin, as key contributors to enhanced seedling development. Furthermore, a positive relationship between sulfur content and glucosinolate levels suggests that sulfur concentration could serve as a useful quality marker for assessing the bioactivity of broccoli-based biostimulants. This study underscores the potential of broccoli-derived extracts as sustainable biostimulants for improving germination and seedling development in pak choi. The findings highlight the influence of seasons on the bioactive composition of extracts, with low temperatures and high relative humidity favoring the accumulation of secondary metabolites and an optimal nutrient balance in plants.

## 1. Introduction

In the contemporary context, there is an imperative for the implementation of valorization and recycling alternatives for all by-product materials generated through agricultural production processes [1]. However, a significant barrier still exists when it comes to translating scientific knowledge into technical application. In response to significant agro-waste generation and in line with the emerging trend of biostimulant application, the use of this material as a source of phytochemicals is a solution that is both practical and viable. Specifically, broccoli stands out as a promising crop due to its high yield and rich bioactive compound profile, making it a valuable resource for the elaboration of biostimulants.

The large-scale production of broccoli (*Brassica oleracea* L. var. *italica*) and cauliflower generates substantial agricultural by-products, with global production exceeding 25.84 million tons in 2021 (FAOSTAT, 2023). Furthermore, although it is a mild-cool season annual plant, its relatively short growing time allows farmers to obtain multiple harvests per year under a Mediterranean climate, enhancing its economic yield [2]. Nevertheless, commercial production focuses primarily on the florets and upper stems, leaving substantial by-products such as lower stems, leaves, and petioles. Previous studies, such as Liu et al. (2018) [3], have pointed out the composition of leaves and stems of broccoli “*Gipsy”* cultivar. In this work, the study of secondary metabolites has demonstrated that broccoli leaves show elevated concentrations of glucosinolates (GSLs), such as hydroxyglucobrassicin and neoglucobrassicin, and phenolic compounds. GSLs are defined as key secondary metabolites in Brassica plants. They are essential for defense against biotic and abiotic stresses. Upon tissue damage, they are enzymatically converted into bioactive products. These compounds trigger resistance mechanisms against environmental stresses [4,5]. On the other hand, phenolic acids are included in the secondary metabolism of broccoli. Among them, caffeic and sinapic acids, which are the main ones, have been related to their defense mechanisms and overall plant health [6]. These phenolic acids possess antioxidant properties that help protect the plant from oxidative stress caused by environmental factors such as UV radiation, drought, and pathogens [7].

As previously mentioned, plant-derived biostimulants can be defined as complex products whose function is to enhance or benefit diverse physiological processes, such as nutrient uptake and efficiency, tolerance to abiotic stress and crop quality [8]. Furthermore, biostimulants can be applied at various stages of plant development, including germination, vegetative growth, flowering, and fruit production [9]. Recently, there has been a growing interest in the utilization of biostimulants during the germination stages in order to reduce the nursery time [10]. In this way, it has been demonstrated that extracts derived from broccoli by-products could enhance the growth of broccoli seedlings by reducing oxidative stress and regulating essential metabolic pathways [11]. However, despite these findings, several aspects related to the variability of broccoli extracts and their bioactive potential remain unexplored. It is essential to consider the impact of sowing time, growth phase and seasonal variation on the secondary metabolism of these broccoli by-products, in order to determine the composition of the subsequent biostimulants that will be industrially produced. Previous studies have found that the highest concentrations of GSLs were observed in field conditions during spring, in contrast to autumn and winter [2]. Additionally, it has been demonstrated that the age of the plant significantly affects the accumulation of metabolites, indicating that the stage of development, as well as seasonal timing, should be carefully considered to optimize the nutritional and functional profile of broccoli [12]. In summary, the bioactive profile and biostimulant potential of broccoli extracts are shaped by the morphology of different tissues, their biochemical composition, and seasonal environmental conditions. Understanding these seasonal variations is essential for optimizing the use of broccoli by-products as bioactive extracts. Moreover, previous studies have largely focused on individual compounds, overlooking the interactions between macro- and micronutrients and secondary metabolites. This gap limits our understanding of how these components work synergistically to enhance biostimulant activity.

Pak choi (*Brassica rapa* subsp. *chinensis* L.) is an under-studied brassica crop, but increasingly valued in the market for its nutritional benefits and agricultural potential. Also known as bok choy, it is a widely cultivated leafy vegetable, particularly in Asia, where it is an essential component of the diet due to its rich content of vitamins, minerals, and antioxidants. Its adaptability to various environmental conditions and short cultivation cycle makes it a highly efficient crop for sustainable agricultural practices [13,14]. Unlike Arabidopsis, which is widely used in research but has limited agronomic relevance, pak choi represents a commercially important crop with practical applications, providing an effective model for evaluating the potential benefits of plant-derived extracts [15].

For all these reasons, the main hypothesis of our study is that broccoli-derived extracts from leaves, stems, and petioles of plants cultivated under three different environmental conditions (temperature and humidity) during autumn, winter, and spring in greenhouse conditions would exert a different biostimulant activity on pak choi seed germination. It was further anticipated that seasonal variations and plant tissues would influence the mineral content and secondary metabolite profiles of the extracts, thereby affecting their biostimulant potential. Addressing the identified gaps, the aim of this study is to analyze the relationship between nutrients, secondary metabolites, and seedling development, in order to provide insights into the potential formulation of customized extracts with enhanced efficacy.

## 2. Materials and methods

### 2.1. Plant material and growth conditions

#### 2.1.1. Plant description.

The study was conducted on broccoli (*Brassica oleracea* L. var. *italica* ‘Ulises’). Seedlings were initially grown in nurseries (Semilleros del Sureste S.A. and Semilleros Jimenado S.A.) for approximately 1.5 months before being transplanted to a greenhouse. The growth of these transplanted plants was studied across three different seasons: autumn (22^nd^ September to 4^th^ December), winter (22^nd^ January to 1^st^ April), and spring (18^th^ March to 3^rd^ June).

#### 2.1.2. Growth conditions.

**Fig 1:**
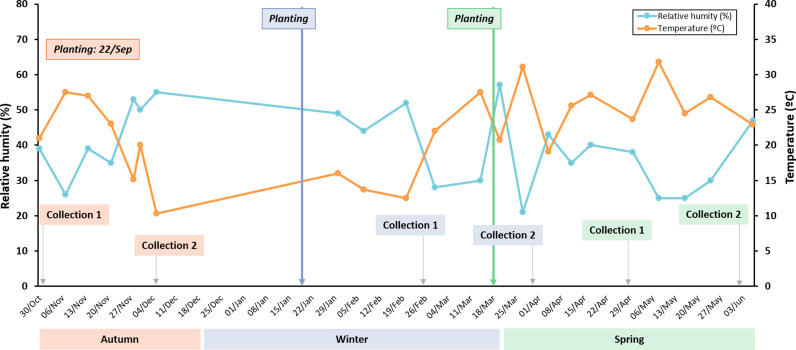
Seasonal climate conditions during broccoli cultivation. Relative humidity (%, blue) and temperature (^o^C, orange) conditions during the plant growth season experiment (autumn, orange; winter, blue; spring, green).

The experiment took place in a greenhouse at the CEBAS-CSIC Experimental Farm in Santomera (Region of Murcia, Spain), under Mediterranean climate conditions [16] (Fig 1). Prior to planting, the substrate (Golden Grow HP Balance EW EP; Projar, Valencia, Spain) was prehydrated for 24 h with water. Once transplanted, seedlings were arranged in three lines of nine plants each and irrigated four times a day (8:00–8:05 am, 12:00–12:15 pm, 4:00–4:05 pm, and 8:00–8:15 pm) with Hoagland solution using 3 L h ⁻ ¹ droppers.

Plant sampling was performed at two time points: 1.5 months and 3 months after planting. The selection of plants for each collection was done randomly, resulting in three randomized groups of three plants each. Once collected, the aerial part of the plants was weighed to determine the fresh weight (FW). A representative portion of this material was separated, weighed, and then dried at 70°C for 5 days to determine its dry weight (DW) and nutrient content. The total dry weight of the aerial part was estimated using a proportionality rule based on the fresh-to-dry weight ratio of the sampled material. The remaining plant material was separated into leaves, stems, and petioles and stored at -80°C for subsequent freeze-drying and extract preparation.

### 2.2. Extraction procedure

Extracts were obtained by mixing 20 g of lyophilized powder with 70% methanol in water in a 1:10 proportion (w:v) following the protocol described by Albaladejo-Marico et al. (2024) [11]. Then, samples were placed in a water bath at 72^o^C for 30 min, with vortexing every 10 min. After that, samples were cooled on ice for 15 min and centrifuged at 10,000 *xg* for 20 min in order to remove fiber and debris. To remove the methanol used in the extraction, a rotary evaporator was employed. Concentrated extracts were collected and filtered through a 0.22 µm pore diameter PVDF Millex Syringe Filter, Durapore (PVDF) by Merck Millipore (Billerica, MA, USA).

### 2.3. Broccoli extracts characterization

#### 2.3.1. Phenolic acids and glucosinolates quantification.

The analysis of GSLs and phenolic compounds was performed using HPLC-ESI-MS at the Metabolomic and Proteomic Laboratory of Murcia University (ACTI-Murcia, Spain). Analyses were performed using a negative ion mode on an Agilent 1290 Series II HPLC system, coupled to an Agilent iFunnel 6550 quadrupole time-of-flight (QTOF) mass spectrometer (Agilent Technologies, Santa Clara, CA, USA). Determinations were performed under the conditions indicated by Albaladejo-Marico et al. (2024) [11]. Three biological replicates were analyzed. For quantification, external standards were used including glucoraphanin (GRA) and glucobrassicin (GBA) (Phytoplan) for GSLs, and chlorogenic acid, caffeic acid and sinapic acid (Sigma-Aldrich) for phenolic compounds.

#### 2.3.2. *Mineral composition.*

Mineral composition was analyzed from both the fresh material and the obtained extracts. Analyses were performed at the Ionomics Laboratory (CEBAS-CSIC, Murcia, Spain) as described in detail in Nicolas-Espinosa et al. (2024) [17] by inductively coupled plasma optical emission spectrometry (ICP-OES).

### 2.4. Seed growth and treatment in vitro

Pak choi (*Brassica rapa ssp. Chinensis L.* Mei Qing Choi) seeds were used to perform germination tests with the extracts, following the method described by Albaladejo-Marico et al. (2024) [11]. All germination tests were conducted under a horizontal laminar flow hood. First, seeds were sterilized with 1:1 sodium hypochlorite:water (NaClO:H2O, v:v) for 10 min. After that, they were rinsed 3 times with Milli-Q water. Then, seeds were placed in sterile 48-well plates (flat bottom, polystyrene, with lids), with one seed per well, containing 500 µ L of 0.8% agar-water medium (PhytoAgar). The plates were then placed in a growth chamber for 8 days. The conditions included a 16-hour light/8-hour darkness cycle, with temperatures 25°C and 20°C, respectively. The relative humidity was set at 60% during the day and 80% during the night, and the photosynthetically active radiation (PAR) was maintained at 400 µmol m^−2^ s^−1^ provided by LEDs (Pacific LED, WT 470 C, LED8OS/840 PSD WB L1600 lights, Philips, Amsterdam, The Netherlands). To determine the effective dose or possible toxicity of the treatment, 1:40, 1:80 and 1:160 dilutions of the extracts were tested.

### 2.5. Statistical analysis

Statistical analyses and data presentation were conducted using Origin (Pro), Version 2024 (OriginLab Corporation, Northampton, MA, USA) and R Studio software (R Core Team 2018). Tests were preceded by a normality test and a Grubbs’ test to identify potential outliers. All data were subjected to a one-way ANOVA followed by post hoc Tukey´s test. All presented values represent means ± SE. R packages FactoMineR [18] and factoextra [19] were used to generate Principal Component Analysis (PCA); and corrplot [20] and psych [21] were used to correlation analysis and plots.

## 3. Results

### 3.1. Broccoli plant growth over the seasons: biomass and mineral content

#### 3.1.1. Biomass analysis.

**Fig 2:**
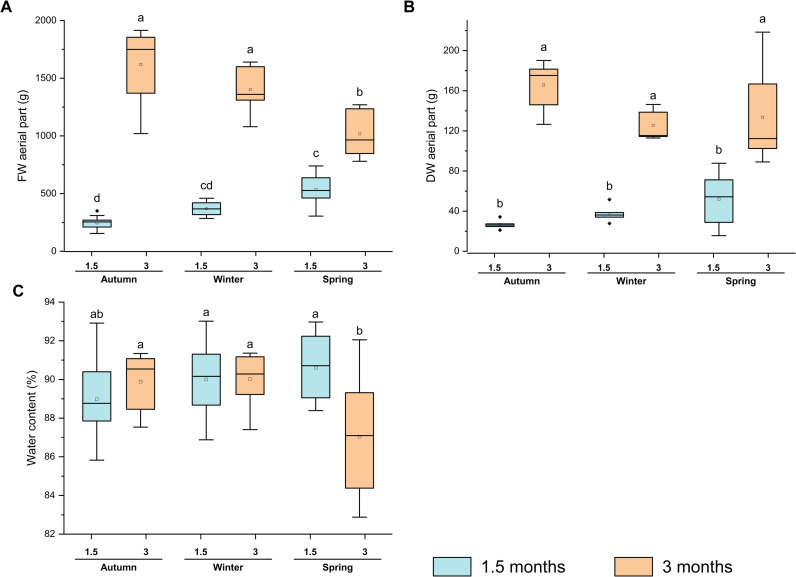
Broccoli biomass through seasons. **(A)** Fresh weight (FW, g), **(B)** dry weight (DW, g), and **(C)** water content (WC, %) of aerial part of broccoli plants at different harvesting points (1.5 and 3 months) and seasons (autumn, winter, and spring). Data are mean ± SE (n = 9-12 for FW and n = 6 for DW). Different letters indicate significant differences according to one-way ANOVA (p < 0.05) followed by Tukey’s post hoc test.

The total biomass of the aerial part of the broccoli plants, was measured at various harvesting times (Fig 2). In terms of FW, significant differences were observed between seasons. At 1.5 months, plants collected in spring exhibited a higher FW. However, at 3 months, the highest FW was recorded in autumn and winter, while spring plants showed a reduced increase in biomass over time (Fig 2A), which correlates with spring environmental conditions (higher temperature and lower RH, Fig 1), which may have limited further biomass accumulation in later stages. In contrast, DW showed no significant seasonal differences (Fig 2B), indicating that the variations observed in FW were primarily due to differences in water content rather than actual biomass accumulation. Water content varied depending on the season and plant age (Fig 2C). In autumn and winter, water content remained stable, showing no significant differences between 1.5- and 3-month-old plants. However, in spring, a decrease was observed in 3-month-old plants, which showed significantly lower water content compared to younger plants and to those from other seasons.

#### 3.1.2. Mineral content analysis.

**Fig 3:**
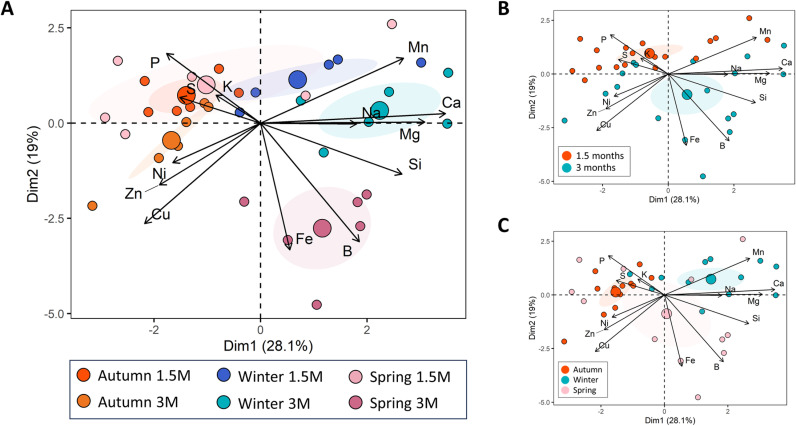
Nutrient profile of broccoli plants through seasons. Principal Component Analysis (PCA) of nutrients in the aerial part of broccoli plants at the different harvesting points (1.5 and 3 months) and seasons (autumn, winter, and spring). Samples are grouped by **(A)** harvest season and development stage, **(B)** by development stage, and **(C)** by season. Arrows indicating loadings of each nutrient; 95% confidence ellipses were plotted for each treatment, each small circle represents the data from an individual sample, while the large circle represents the centroid of mean values.

The mineral content of the plant material (aerial part of broccoli plants) was also analyzed (Fig 3 and S1 Table). The Principal Component Analysis (PCA) displays the main differences between the groups in terms of nutrients. Fig 3A shows that the group that is most different from the rest the one that encompasses the plants harvested in spring with 3 months. In general, the greatest separation is due to the time factor, clearly separating the groups of samples taken at 1.5 months and 3 months, the latter being at the bottom of the graph and showing a positive correlation with the micronutrients B and Fe. While the younger plants correlate positively with P and K (Fig 3B). As for the seasons, it was found that autumn and winter were clearly separated, while the samples taken in spring were positioned between the other two seasons (Fig 3C).

### 3.2. Characterization of secondary metabolite-enriched extracts from different parts of broccoli plants

**Fig 4:**
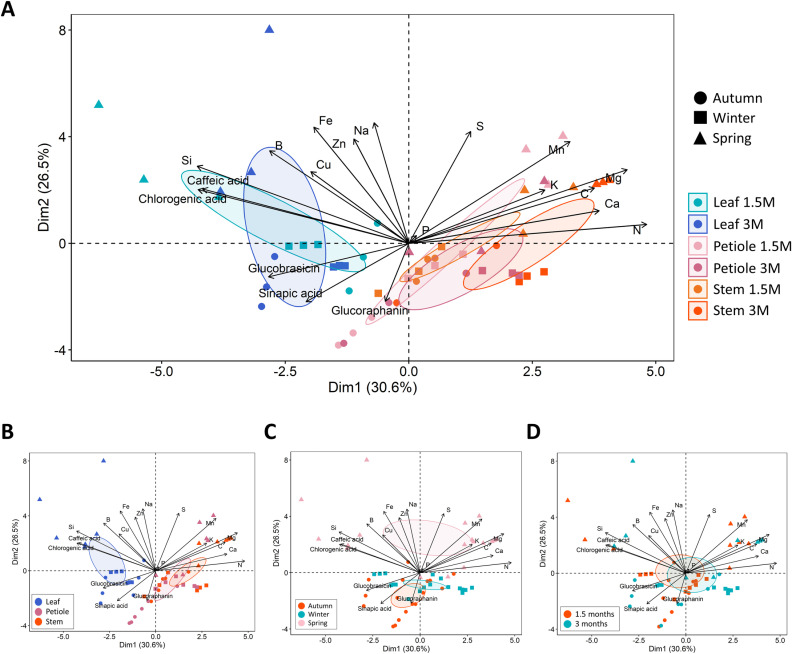
Nutrient and secondary metabolite profile of broccoli extracts. Principal Component Analysis (PCA) of nutrients and metabolites in broccoli extracts made with leaf, petiole, and stem from plant collected in autumn, winter and spring at two development stages (1.5 and 3 months). Samples are grouped **(A)** by part of plant and development stage, **(B)** by part of plant, **(C)** by season, and **(D)** by development stage. Arrows indicating loadings of each nutrient; 95% confidence ellipses were plotted for each treatment, each small circle represents the data from an individual sample, while the large circle represents the centroid of mean values.

**Fig 5:**
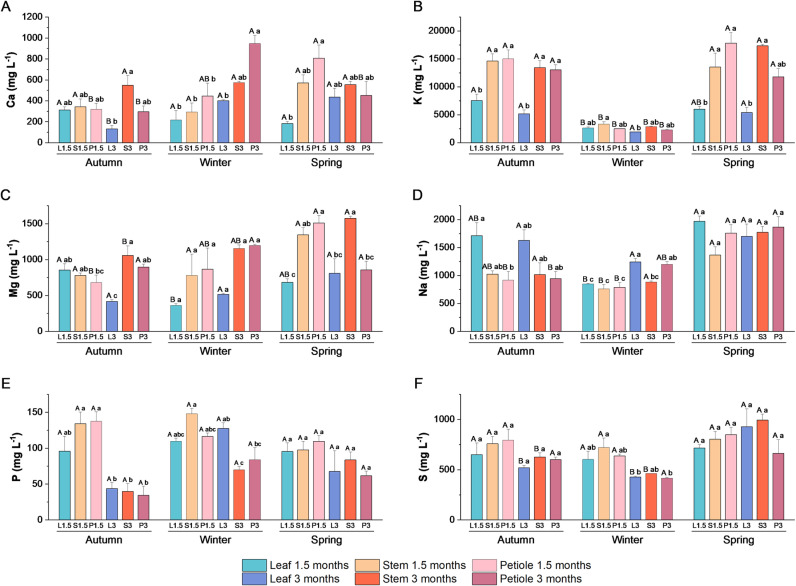
Macronutrients analysis of broccoli extracts. Macronutrients analysis in broccoli extracts made with leaf, petiole, and stem from plant collected in autumn, winter and spring at two development stages (1.5 and 3 months). **(A)** Ca (mg L^-1^), **(B)** K (mg L^-1^), **(C)** Mg (mg L^-1^), **(D)** Na (mg L^-1^), **(E)** P (mg L^-1^), and **(F)** S (mg L^-1^). Data are mean ± SE (n = 3-6). Capital letters indicate significant differences according to one-way ANOVA (p < 0.05) followed by Tukey’s post hoc test performed for each extract through the seasons. Lower case letters indicate differences according to one-way ANOVA (p < 0.05) followed by Tukey’s post hoc test performed within each season to compare different type of extracts.

**Fig 6:**
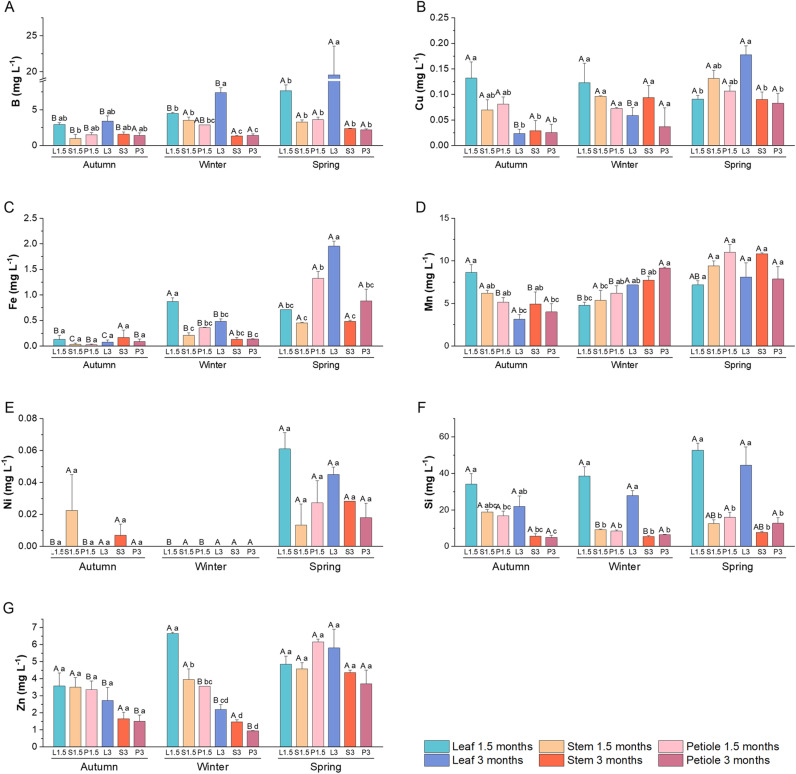
Micronutrients analysis of broccoli extracts. Micronutrients analysis in broccoli extracts made with leaf, petiole, and stem from plant collected in autumn, winter and spring at two development stages (1.5 and 3 months). **(A)** B (mg L^-1^), **(B)** Cu (mg L^-1^), **(C)** Fe (mg L^-1^), **(D)** Mn (mg L^-1^), **(E)** Ni (mg L^-1^), **(F)** Si (mg L^-1^), and **(G)** Zn (mg L^-1^). Data are mean ± SE (n = 3-6). Capital letters indicate significant differences according to one-way ANOVA (p < 0.05) followed by Tukey’s post hoc test performed for each extract through the seasons. Lower case letters indicate differences according to one-way ANOVA (p < 0.05) followed by Tukey’s post hoc test performed within each season to compare different type of extracts.

**Fig 7:**
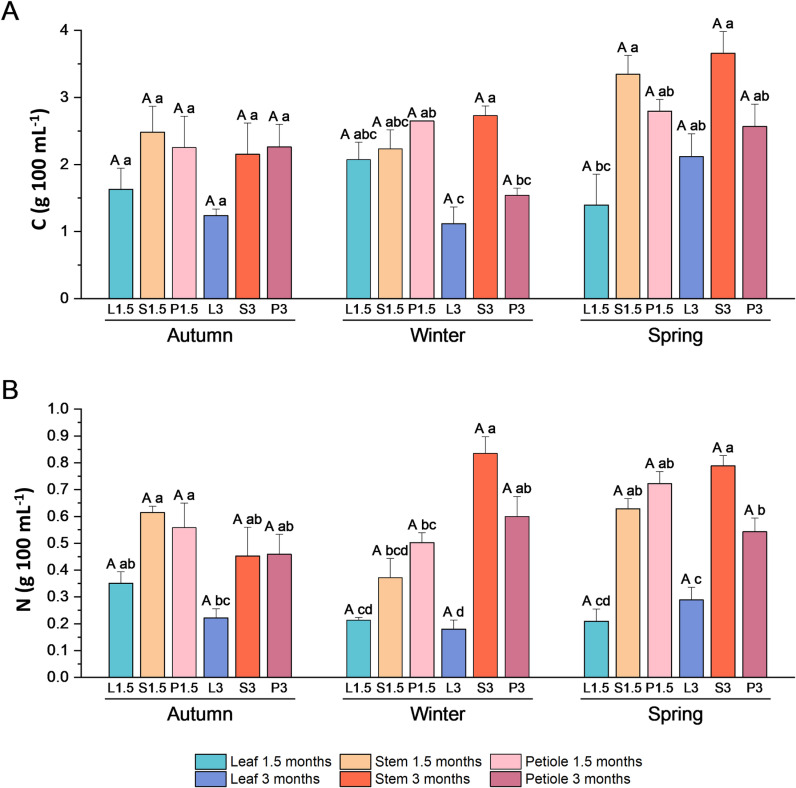
Carbon and nitrogen analyis of broccoli extracts. **(A)** Carbon (C) and **(B)** nitrogen (N) content (g 100 mL^-1^) in broccoli extracts made with leaf, petiole, and stem from plant collected in autumn, winter and spring at two development stages (1.5 and 3 months). Data are mean ± SE (n = 3-6). Capital letters indicate significant differences according to one-way ANOVA (p < 0.05) followed by Tukey’s post hoc test performed for each extract through the seasons. Lower case letters indicate differences according to one-way ANOVA (p < 0.05) followed by Tukey’s post hoc test performed within each season to compare different type of extracts.

Extracts enriched in secondary metabolites obtained from different aerial parts of broccoli plants (leaves, stems, and petioles) collected in different seasons were analyzed to determine their mineral, GSLs, and phenolic composition (Fig 4–7).

The PCA analysis provides a global overview of the distribution of groups based on the analyzed variables. The first two principal components (PC1 and PC2) accounted for 57.1% of the total variance. Mineral content, phenolic compounds, and GSLs contributed significantly to the group separation. The analysis revealed correlations between secondary metabolites and minerals such as Si, B, and Cu, which are associated with leaf extracts clustering on the left side of the plot. In contrast, macronutrients and essential elements (S, Mg, Ca, K, C, N) were positioned in the upper right quadrant, suggesting a stronger correlation with extracts obtained from petioles and stems. This differentiation proposes that the biochemical composition of the extracts is closely linked to the plant organ used, reflecting distinct metabolic and nutritional profiles (Fig 4A).

Specifically, leaf extracts differed from petiole and stem extracts in terms of mineral and secondary metabolite content (Fig 4B), with a strong correlation between leaves and caffeic and chlorogenic acids. Additionally, samples from autumn and winter clustered together, whereas spring samples formed a distinct group. Notably, GSLs and sinapic acid showed a stronger association with samples from autumn, while phenolic compounds, such as caffeic and chlorogenic acids, were more prevalent in spring (Fig 4C). In contrast, no separation was observed in the overall composition of the extracts based on plant age (1.5 vs. 3 months) (Fig 4D).

#### 3.2.1. Mineral content.

Fig 5 presents the concentration of each macronutrient in all the extracts obtained in the framework of the present study. The concentration of Ca exhibited fluctuations throughout the seasons, displaying higher levels in spring in the extract obtained from 1.5-month-old petioles and higher levels in winter in the 3-month-old petiole extract. Additionally, notable variations were observed in leaf Ca content at the three-month stage, with the lowest calcium content occurring during the autumn season (Fig 5A). The K content typically declines during the winter season. The extracts with the highest potassium content were those obtained from the stem and petiole in autumn and spring (Fig 5B). The extracts with the highest Mg content are those obtained in spring with the petiole of 1.5 months, while the lowest values were found in leaf extracts (Fig 5C). The Na levels are typically higher in extracts obtained in spring, in autumn in leaf extracts and in winter with leaves at 3 months (Fig 5D). With regard to P, significant differences were only identified in autumn extracts, where P levels were lower at 3 months compared to the 1.5 autumn extracts (Fig 5E). Finally, the highest S levels were found in the extracts made from leaves and stems of plants grown for 3 months in spring (Fig 5F).

The micronutrient content of the extracts obtained is shown in Fig 6. Extracts made with leaves have the highest B content, which is higher in those made with material harvested in spring (Fig 6A). The highest Cu contents were found in extracts made from leaves harvested at 3 months in spring, (Fig 6B). The lowest Fe contents were found in all autumn extracts, while the extracts with the highest Fe contents were those made with 1.5-month-old leaves in winter and those made in spring, especially with 3-month-old leaves (Fig 6C). As for Mn, few changes were found and it should be noted that in autumn the extract with the highest content was that of the 1.5-month-old leaf (Fig 6D). The Ni content in the extracts was generally very low in all extracts, being slightly higher in the spring extracts (Fig 6E). Si levels were higher in the leaf extracts, with levels remaining constant throughout the seasons (Fig 6F). Finally, Zn levels were constant between the different seasons, with a higher concentration observed in the 3-month petiole extract in spring compared to the other seasons (Fig 6E).

The C and N contents did not show any significant differences between the different seasons (Fig 7). When comparing extracts derived from different parts, the lowest C levels were observed in leaf and petiole extracts at three months of winter (Fig 7A). With regard to N content, the lowest levels were identified in leaf extracts (Fig 7B).

#### 3.2.2. Secondary metabolites content: glucosinolates and phenolic compounds.

**Fig 8:**
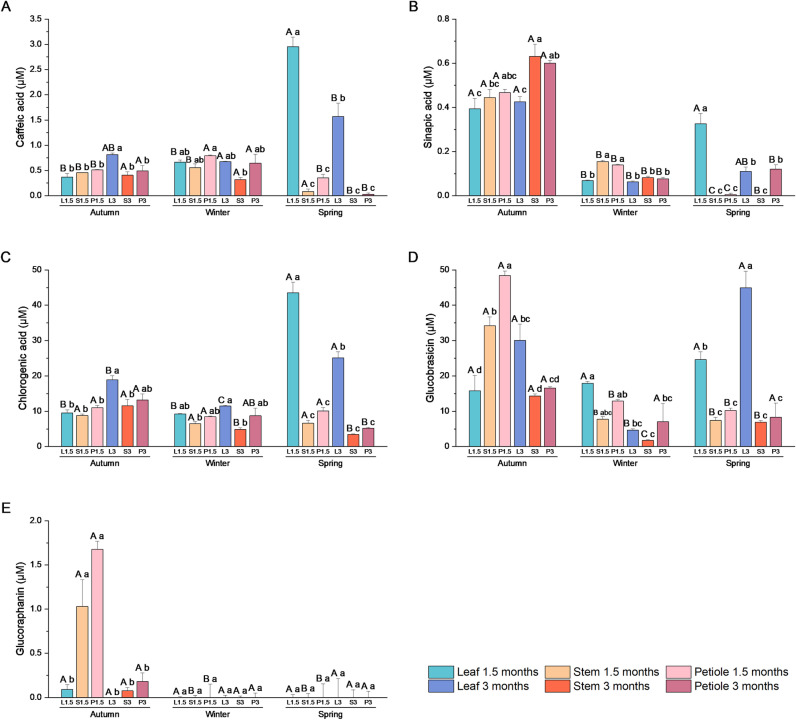
Secondary metabolites content of broccoli extracts. Phenolic and glucosinolates in broccoli extracts made with leaf, petiole, and stem from plant collected in autumn, winter and spring at two development stages (1.5 and 3 months). **(A)** Caffeic acid (µM), **(B)** sinapic acid (µM), **(C)** chlorogenic acid (µM), **(D)** glucobrassicin (µM), and **(E)** glucoraphanin (µM). Data are mean ± SE (n = 3). Capital letters indicate significant differences according to one-way ANOVA (p < 0.05) followed by Tukey’s post hoc test performed for each extract through the seasons. Lower case letters indicate differences according to one-way ANOVA (p < 0.05) followed by Tukey’s post hoc test performed within each season to compare different type of extracts.

The phenolic compounds and GSLs present in the extracts were analyzed by HPLC (Fig 8). The caffeic acid content of each extract remains stable throughout the autumn and winter seasons, exhibiting in spring a notable decline in the stem and petiole extracts and a substantial increase in the leaf extracts, reaching levels between 2 and 3 µM(Fig 8A). With regard to sinapic acid, the highest levels are observed in all extracts prepared with material from the autumn harvest. Among these, the leaf extracts exhibit the lowest levels (Fig 8B). The pattern observed for chlorogenic acid is similar to that of caffeic acid, with the highest levels present in leaves, particularly at three months during the autumn period and at one and a half and three months during the spring period. Conversely, the lowest levels of this compound are typically found in stem extracts, occurring at three months during the winter and spring periods (Fig 8C). The content of GSLs, including glucobrassicin and glucoraphanin, was analyzed. Fig 8D illustrates that the highest levels of glucobrassicin are present in the petiole extracts from plants that were 1.5 months old in the autumn, the leaf extracts from plants that were 3 months old in the autumn and spring, and the stem extracts from plants that were 1.5 months old in the autumn. The lowest levels are typically observed in winter and spring extracts, particularly in those derived from stems and petioles. Finally, the highest concentration of glucoraphanin was observed in the 1.5-month-old stem and petiole of autumn extracts (Fig 8E).

#### 3.2.3. Correlation of extract glucosinolates concentration and plant sulfur.

**Fig 9:**
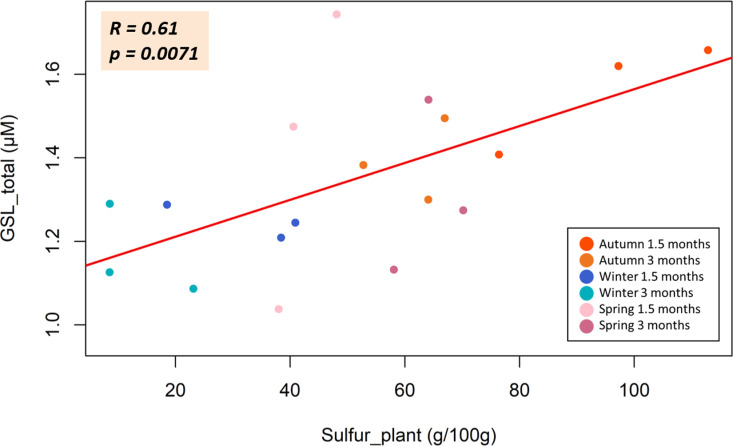
Glucosinolates and sulfur correlation. Relationship between sulfur content in broccoli plants (Sulfur_plant, g/100g) and total glucosinolate concentration (GSL_total, µ M) in different seasons (autumn, winter, and spring) and plant ages (1.5 and 3 months) indicating, with Pearson’s correlation coefficient (R) and p-value (p). Data points are color coded according to season and age: autumn (red and orange), winter (dark and light blue) and spring (pink and purple).

Fig 9 depicts the correlation between the sulfur content of the broccoli plant and the total GSLs content in the extracts. The data points are color-coded according to season and plant age: autumn (red-orange), winter (blue), and spring (pink). A positive correlation between sulfur content and total GSLs levels is observed, with a Pearson correlation coefficient of *R*= 0.61 and a significance level of *p* = 0.0071. This indicates a statistically significant relationship, suggesting that higher sulfur content in the plant is associated with an increase in GSLsaccumulation.

### 3.3. Effect of broccoli extracts on pak choi germination

#### 3.3.1. Weight of pak choi seedling treated with broccoli extracts.

**Fig 10:**
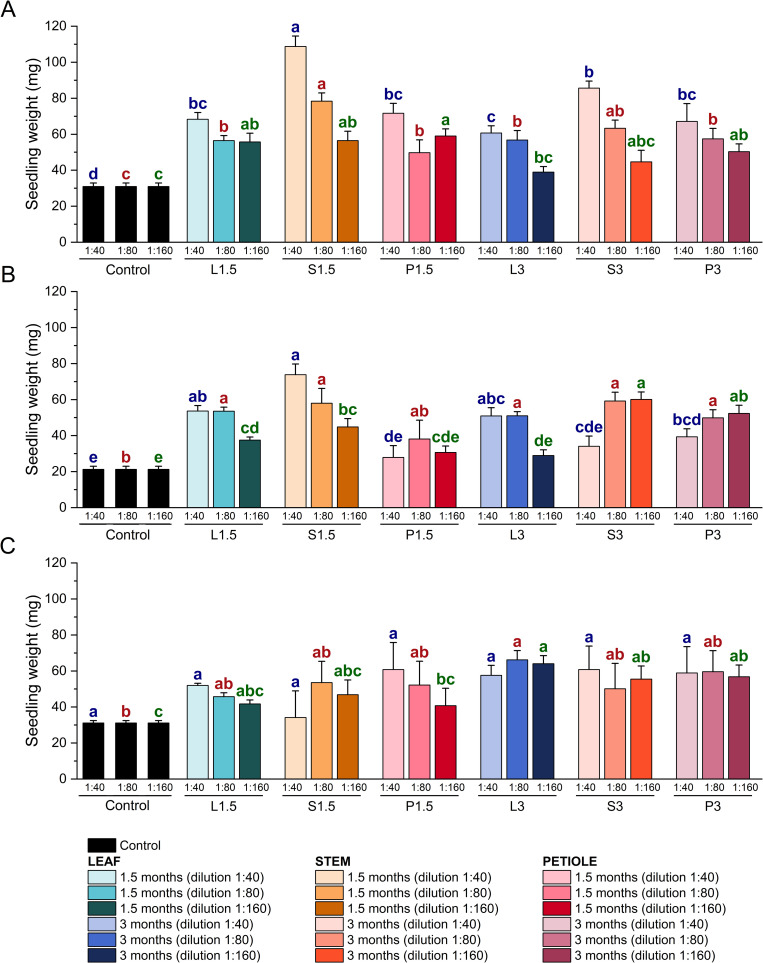
Effect of broccoli extracts on pak choi seedling germination. Weight (mg) of pak choi seedling grown in presence of different dilutions (1:40, 1:80, and 1:160) of extracts obtained from leaf (L), stem (S) and petiole (P) of broccoli plants collected at two development stages (1.5 and 3 months) in three different seasons: **(A)** autumn, **(B)** winter, and **(C)** spring. Data are mean ± SE (n = 5-12). Different letters indicate significant differences according to one-way ANOVA (p < 0.05) performed within each dilution followed by Tukey’s post hoc test. Each control bar corresponds to the dilution 1:40 (blue letters), 1:80 (red letters), and 1:160 (green letters), respectively. The Tukey’s post hoc test has been performed to compare bars of different colors (different types of extract) at the same dilution.

All extracts were tested at three different dilutions of the original extracts (1:40, 1:80, and 1:160) as biostimulants on pak choi seed germination and sprout development (Fig 10 and S1B Fig). The results showed that, in general, all types of extracts produced a significant increase in sprout biomass compared to the control. Among the extracts obtained with plant material harvested in autumn, the extract made with 1.5-month-old stems stood out, as it produced an increase of about 250% compared to untreated seeds (Fig 10A). Furthermore, in these extracts we see a dilution-dependent effect, the more diluted the extract, the smaller the effect. This is no longer the case for all the winter extracts, where we find that the stem extract 3 months and the petiole extract 1.5 and 3 months do not meet this requirement. As for the winter extracts, it would also highlight the 1.5 stem extract at the highest dilution, (Fig 10B). Finally, with the extracts obtained from spring harvested material, when applied to pak choi seeds, we found that with the more concentrated extracts (1:40) there was no significant difference between the weight of the treated pak choi shoots and the controls. At a higher dilution (1:80), the leaf extract promoted a significant increase in shoot biomass at three months. Finally, at a dilution of 1:160, leaf, stem and petiole extract at 3 months also resulted in a significant increase (Fig 10C).

#### 3.3.2. An overview of the influence of the extract on the seedling weight.

**Fig 11:**
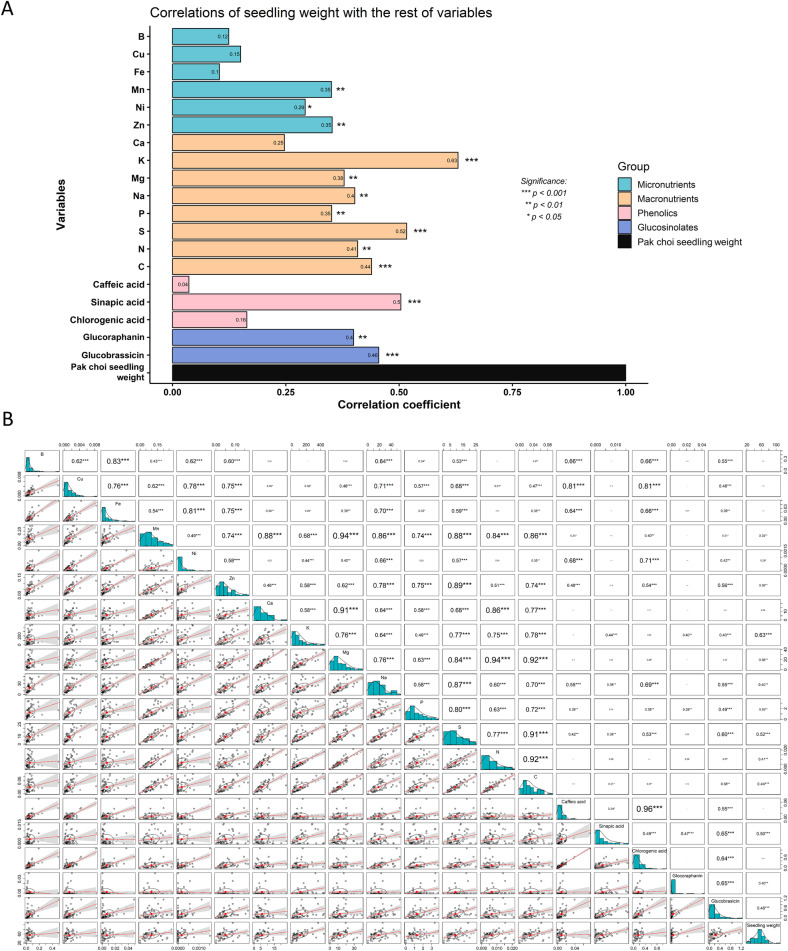
Overall correlation analysis of broccoli extracts components. **(A)** Correlation coefficients of seedling weight with various variables, including micronutrients, macronutrients, phenolics, and glucosinolates. The bar graph shows the strength and direction of correlations, with significance levels indicated: ***p < 0.001, **p < 0.01, *p < 0.05. Blue bars represent micronutrients, orange bars macronutrients, pink bars phenolics, and purple bars glucosinolates. **(B)** Correlation plot among all the variables where the lower triangular part of the correlation matrix displays scatterplots, while the upper triangular part shows Pearson correlation coefficients with significance stars (***p < 0.001, **p < 0.01, *p < 0.05).

An overall correlation analysis shows that pak choi seedlings weight has positive correlations with several compounds present in broccoli extracts: micronutrients, macronutrients, phenolics, and GSLs (Fig 11). Considerably positive correlations were found with two macronutrients, K (*R = 0.63, p < 0.001*) and S (*R = 0.52, p < 0.001*). A positive correlation was also found with the phenolic compound sinapic acid (*R = 0.5, p < 0.001*), as well as with the two GSLs detected in the extracts, glucoraphanin (*R = 0.4, p < 0.01*) and glucobrassicin (*R = 0.46, p < 0.001*) (Fig 11A). Furthermore, Fig 11B illustrates the interrelationships between all the variables, that is, all the components of the extracts. Some of these interrelationships are particularly noteworthy, such as the correlations between chlorogenic acid and Ca and Ni, or the correlation between S with sinapic acid and glucobrassicin. Additionally, it is worth noting that there are strong positive correlations between carbon and nitrogen with all macronutrients.

## 4. Discussion

The seasonal variation in glucosinolate content of field-grown broccoli has been previously reported [2]. Additionally, it has been previously demonstrated that broccoli extracts rich in secondary metabolites promote shoot development while also creating a memory effect in plants, enhancing their growth at later stages [11]. Accordingly, using broccoli by-products from various seasons, it became crucial to determine how this seasonal variability (Fig 1) impacted the composition of extracts derived from broccoli by-products (stems, petioles and leaves). Furthermore, to assess the potential stimulatory effect of these extracts, we analyzed their composition in relation to pak choi germination (S3 Fig 2). This research holds particular relevance for the agricultural industry, as it seeks to standardize germination conditions, optimize sprout size, and accelerate the transition from seedbed to field [22].

In order to gain an understanding of the physiological state of the plants from which the extracts would be obtained, biomass determinations (FW and DW) and nutrient profile analysis of the aerial parts were conducted. While no significant differences in FW were observed between autumn and winter, a notable reduction in FW was recorded in plants harvested after three months in spring. This decline coincided with higher temperatures (25–30°C) towards the end of the experimental period, suggesting that increased temperature-induced water loss may have contributed to biomass reduction. Elevated temperatures accelerate water evaporation, which not only decreases overall water content but also may impair biomass accumulation. Therefore, it is likely that the reduced biomass observed in spring could be partly attributed to water stress, which affects not only the overall water content but also the plants’ ability to accumulate fresh biomass.

A clear distinction in mineral nutrient composition was observed between broccoli plants cultivated in spring and those grown in other seasons, suggesting that elevated temperatures significantly influence both nutrient uptake and overall yield. These enhancements have been reported to be related to temperature-dependent alterations in nutrient transport and absorption, processes that are closely linked to transpiration rates, which tend to increase under high-temperature conditions [23]. Despite the seasonal variation in FW, DW remained stable, indicating that FW differences were primarily due to reduced water content rather than biomass accumulation. The primary nutrient shifts included a decrease in P and an increase in B. Interestingly, B uptake, unlike that of other nutrients, appears unaffected by elevated temperatures. In fact, studies on tomato plants have shown that B uptake does not diminish at high temperatures; instead, it increases at 35°C [24]. A similar trend may explain the higher B levels observed in broccoli plants harvested in spring.

The composition of broccoli extracts obtained was analyzed to evaluate the impact of the components on their activity in stimulating germination. At the nutrient level, leaf extracts showed significant divergence from those derived from petioles and stems. Seasonally, a similar trend to that of the plant was observed, with spring extracts diverging most significantly from those obtained in autumn and winter. However, the overall composition of the extracts was not significantly influenced by the development stage of broccoli plants (1.5 or 3 months). This suggests that seasonal environmental factors, such as temperature and water availability, play a more dominant role than plant age in shaping the nutrient composition of the extracts. The regulation of nutrient uptake is highly responsive to external conditions, which may override developmental effects. Additionally, the early establishment of metabolic patterns in broccoli could explain the negligible influence of plant age on extract composition.

With regard to the secondary metabolites examined in the extracts, it is noteworthy that an increase in GSLs and phenolics was observed in the samples derived from the material obtained in the spring season, particularly in the leaves, both at 1.5 months and at 3 months. This may be due to the fact that these metabolites are associated with defense mechanisms against abiotic stresses, such as high temperatures [25], and against biotic stresses, such as pathogens [26]. Indeed, higher temperatures generally promote the proliferation and spread of plant pathogens [27]. Therefore, plants would increase the production of defensive compounds to protect themselves and accumulate them in the leaves, as they are the main photosynthetic and metabolically active organs, which are particularly susceptible to pathogen attack.

Moreover, although the extraction process enriched phenolic compounds and GSLs, very low levels of glucoraphanin were detected in the winter and the spring samples. These samples were obtained from plants grown under conditions of higher temperature stress than those obtained from autumn samples. It is probable that the plant was degrading compounds such as glucoraphanin to yield their degradation products, which are distinguished by heightened bioactivity relative to the GSLs themselves [4]. This hypothesis aligns with the findings of a study conducted under drought conditions, which revealed that the accumulation of aliphatic GSLs did not directly enhance stress tolerance. However, increasing the content of GSL degradation products, such as ITCs, may contribute to enhancing tolerance [28]. In this way, the degradation product of glucoraphanin, sulforaphane, may be produced in the aerial part of broccoli plants as a defense mechanism against temperature stress.

Regarding germination-stimulating activity, extracts obtained in autumn and winter exhibited the strongest effects, whereas those from spring had a more limited impact (Fig 10 and S2 Fig 1B). Autumn extracts contained higher concentrations of secondary metabolites, whereas spring extracts showed elevated levels of micro- and macronutrients (S2 Fig 1A). However, the increased macronutrient levels in spring extracts had a comparatively lower influence on seedling development. Notably, these treatments also demonstrated a high concentration of chlorogenic acid and boron. Recently, it has been reported that plants are capable of generating chlorogenoborate complexes, which may have been formed as a result of these high concentrations. Since these complexes have not been extensively investigated, their elevated concentrations could potentially exert a detrimental impact on plant growth [29].

Winter extracts, by contrast, displayed a balanced composition of these components. Excessive mineral nutrients in the growth medium can induce oxidative and osmotic stress in seeds and seedlings, as high nutrient levels can alter root-zone pH, ultimately significantly reducing shoot growth, as demonstrated in previous studies [30]. Interestingly, the most beneficial effects of spring extracts were observed at lower dilutions, suggesting that reduced nutrient concentrations may promote growth even in the presence of fewer secondary metabolites. This could be explained by a hormetic effect, where low concentrations of certain nutrients act as biostimulants, while excessive levels may induce osmotic stress and negatively impact germination [31]. Additionally, some secondary metabolites, such as phenolics and GSLs, may exert inhibitory or allelopathic effects at high concentrations, which could limit germination and seedling growth [32]. Thus, the optimal balance between nutrient availability and metabolite concentration in diluted extracts likely enhances germination by minimizing potential stressors while still providing essential growth-promoting elements.

Several macro- and micronutrients present in the extracts demonstrated a positive correlation with seedling weight, indicating that these elements exert a biostimulant effect at appropriate concentrations (Fig 11). Notably, K and S contents of the extracts were strongly correlated with each other. K is considered essential to ensure optimal plant growth as it is an activator of enzymes involved in protein synthesis, sugar transport or C and N metabolism [30]. Similarly, S is an essential nutrient, as it serves as a structural component of proteins, vitamins, and cofactors required for proper plant growth and development. In fact, a large amount of S is required during seed development [33] and in a study where an external source of additional S was applied to bean, pea, wheat, and maize seeds, germination success and seedling size increased while the time to germination decreased [34].

When comparing different aerial tissues as leaf, stem and petiole, the best results in autumn and winter were obtained from stem extracts, while in spring, extracts from 3-month-old leaves showed superior efficacy. These leaves contain lower levels of macronutrients but are rich in glucobrassicin and sinapic acid, which may explain their growth-stimulating effects. This aligns with our previous findings that glucobrassicin promotes shoot growth [11]. The correlation analysis supports this, showing a strong positive relationship between GSLs and phenolic compounds in the extracts and seedling weight, highlighting the significant role of secondary metabolites in enhancing growth (Fig 11). Specifically, sinapic acid exhibits a strong correlation with shoot weight and is most abundant in the autumn extracts, which have shown the most pronounced biostimulant effect. Recent research has explored the biostimulant potential of phenolic compounds, showing that these compounds, when applied either foliarly or through the roots, can increase biomass and photosynthesis in mature plants [35,36], as well as promote germination, rooting, sprouting [32], and adventitious roots formation in shoots [37]. It is clear that sinapic acid and glucobrassicin significantly influence the development of pak choi seedlings, as demonstrated by the results with autumn extracts. In contrast, the germination test using spring extracts did not produce similarly positive outcomes. Despite the high levels of sinapic acid, caffeic acid, and glucobrassicin in the leaf extracts, these also contained elevated concentrations of micronutrients such as B, Cu, Fe, Ni, and Si, which may have toxic effects. Moreover, the dose-dependent effect observed when seeds were treated with specific extracts—particularly those from spring samples—appeared to diminish. This could be due to the excessive mineral content in these extracts.

The correlation found between total S content in the aerial parts of broccoli plants and the GSLs content in the extracts is particularly valuable (Fig 9). This relationship suggests that S content could serve as a reliable quality marker for estimating the GSLs levels in extracts derived from broccoli by-products, providing a straightforward analysis tool to assess the bioactive potential of these biostimulants. Given that GSLs may represent up to 30% of the total sulfur content in plants, their accumulation is closely linked to the overall sulfur status of the plant [38]. Studies have demonstrated that sulfur levels are often correlated with GSL content, as shown by the increased GSL concentrations associated with an increase in the sulfur content of the plants, which also depends on the nutritional supply [38]. However, during standard sulfate fertilization (1 mM), the relationship has not been established until now. This insight enables more efficient quality control in the production of GSLs, ensuring consistency and bioactive efficacy across batches by monitoring sulfur as an indirect indicator of GSL content.

Notwithstanding the valuable information provided by this study on the seasonal variability and biostimulant potential of broccoli extracts, certain constraints need to be noted. Firstly, the sample size was limited to specific seasonal conditions and to a single broccoli variety, which may restrict the generalizability of the results to other cultivars or growing conditions. Furthermore, the study concentrated predominantly on the biochemical composition of the extracts and their effects on germination and seedling growth, overlooking other potential physiological effects on plant development. To comprehensively elucidate the underlying mechanisms, further studies incorporating specific metabolomic and transcriptomic analyses are essential. Notwithstanding these limitations, the study underscores significant trends that may inform future research and the development of optimized biostimulant formulations.

In summary, the differential effectiveness of broccoli extracts across seasons can be attributed to the distinct nutrient and secondary metabolite profiles influenced by environmental conditions. Autumn and winter extracts (characterized by low temperatures and high relative humidity), exhibited higher concentrations of secondary metabolites and a balanced nutrient composition, making them more effective biostimulants for seedling growth. In contrast, spring extracts (characterized by high temperatures and low relative humidity), rich in macronutrients, demonstrated limited effectiveness, which may correspond to the fact that broccoli plants were growing less during this season. The elevated mineral nutrient content in spring extracts could also lead to oxidative and osmotic stress at higher concentrations, potentially inhibiting growth. Furthermore, the observed positive correlations between specific compounds, such as sinapic acid and glucobrassicin, and the seedlings growth underscore the significance of secondary metabolites in promoting seedling development. These findings highlight the necessity of considering both nutrient types and concentrations, along with seasonal variability and plant developmental stages, in biostimulant formulation. Additionally, understanding the specific differences between extracts from various plant parts could be key for the elaboration of ‘à la carte’ extracts, which can be customized to contain specific levels of particular nutrients or elements to develop more precise and targeted applications. Finally, the strong correlation between sulfur content and GSLs levels indicates the potential of sulfur determination as a simple quality marker for estimating the bioactive potential of broccoli extracts, aiding in the standardization and optimization of their use as biostimulants.

## Supporting information

S1 Table Mineral content analysis. Nutrients in the aerial part of broccoli plants at the different harvesting points (1.5 and 3 months) and seasons (autumn, winter, and spring). Data are mean ± SE (n = 3-6).
(PDF)

S2 Fig 1 Summary of broccoli extract composition and biostimulant effect. (**A**) Summary of the composition of broccoli extracts obtained from leaves (L), stems (S) and petioles (P) harvested at 1.5 and 3 months in autumn, winter and spring. (**B**) Change (in %) in the biomass of pak choi seedlings treated with the different extracts compared to untreated seedlings. Data are mean ± SE (n = 5-12).
(PDF)

S3 Fig 2 Images of Pak Choi seedlings grown on control agar and on agar with broccoli extracts obtained from leaves (L), stems (S) and petioles (P) harvested at 1.5 and 3 months in autumn, winter and spring. (PDF)

S1 Data “resultados PLOSONE.pdf” File with all the data obtained in this research work. (PDF)
