# Supplementary material for: Valorization of broccoli by-products: seasonal variations in bioactive compounds and their biostimulant effects on pak choi germination
Source: PLoS One. 2025 May 15;20(5):e0323848. doi: 10.1371/journal.pone.0323848 (PMC12101848; doi:10.1371/journal.pone.0323848)
Supplement: S1 Table — Nutrients in the aerial part of broccoli plants at the different harvesting points (1.5 and 3 months) and seasons (autumn, winter, and spring). Data are mean ± SE (n = 3-6). (PDF) [file pone.0323848.s001.pdf]

S1 Table. Nutrients in the aerial part of broccoli plants at the different harvesting points (1.5 and 3 months) and seasons (autumn, winter, and spring). Data are mean ± SE (n=3-6).

	Autumn		Winter		Spring	
	1.5 months	3 months	1.5 months	3 months	1.5 months	3 months
Ca (mg g^{-1} DW)	23.94 ± 1.17	27.41 ± 1.12	32.92 ± 2.77	43.47 ± 2.41	21.85 ± 2.81	29.86 ± 1.33
K (mg g^{-1} DW)	49.84 ± 3.28	33.08 ± 1.4	42.49 ± 0.96	33.06 ± 3.66	48.29 ± 4.9	41.04 ± 2.14
Mg (mg g^{-1} DW)	5.39 ± 0.29	4.52 ± 0.16	5.93 ± 0.35	5.44 ± 0.33	4.55 ± 0.5	6.04 ± 0.35
Na (mg g^{-1} DW)	6.85 ± 0.4	8.23 ± 0.25	7.59 ± 0.57	8.02 ± 0.37	9.29 ± 1.29	8.79 ± 0.58
P (mg g^{-1} DW)	3.58 ± 0.24	3.28 ± 0.19	3.75 ± 0.27	3.19 ± 0.13	2.79 ± 0.28	2.09 ± 0.25
S (mg g^{-1} DW)	15.39 ± 0.44	14.21 ± 0.51	12.7 ± 0.16	11.86 ± 0.51	13.43 ± 1.26	13.2 ± 0.68
B (mg g^{-1} DW)	27.65 ± 1.92	34.51 ± 1.88	28.99 ± 2.27	45.23 ± 4.25	32.03 ± 1.19	79.79 ± 2.5
Cu (mg g^{-1} DW)	1.99 ± 0.08	2.47 ± 0.22	1.92 ± 0.13	1.71 ± 0.14	2.04 ± 0.25	2.36 ± 0.2
Fe (mg Kg^{-1} DW)	72.72 ± 4.36	72.19 ± 4.25	65.39 ± 1.88	72.37 ± 3.71	56.55 ± 5.68	89.26 ± 6.24
Mn (mg Kg^{-1} DW)	120.44 ± 3.53	103.77 ± 3.59	136.56 ± 7.98	145.86 ± 8.62	119.56 ± 10.69	116.05 ± 11.67
Ni (mg Kg^{-1} DW)	0.76 ± 0.16	3.37 ± 0.88	0.25 ± 0.12	0.16 ± 0.02	0.17 ± 0.04	0.55 ± 0.2
Si (mg Kg^{-1} DW)	211.45 ± 8.63	190.93 ± 15.02	344.4 ± 14.25	420.21 ± 9.42	171.36 ± 12.29	397.81 ± 38.01
Zn (mg Kg^{-1} DW)	27.12 ± 1.63	22.28 ± 2.03	21.02 ± 2.21	20.27 ± 1.06	23.61 ± 2.78	29.51 ± 2.74
