# Supplementary material for: Valorization of broccoli by-products: seasonal variations in bioactive compounds and their biostimulant effects on pak choi germination
Source: PLoS One. 2025 May 15;20(5):e0323848. doi: 10.1371/journal.pone.0323848 (PMC12101848; doi:10.1371/journal.pone.0323848)
Supplement: S2 Fig — (A) Summary of the composition of broccoli extracts obtained from leaves (L), stems (S) and petioles (P) harvested at 1.5 and 3 months in autumn, winter and spring. (B) Change (in %) in the biomass of pak choi seedlings treated with the different extracts compared to untreated seedlings. Data are mean ± SE (n = 5-12). (PDF) [file pone.0323848.s002.pdf]

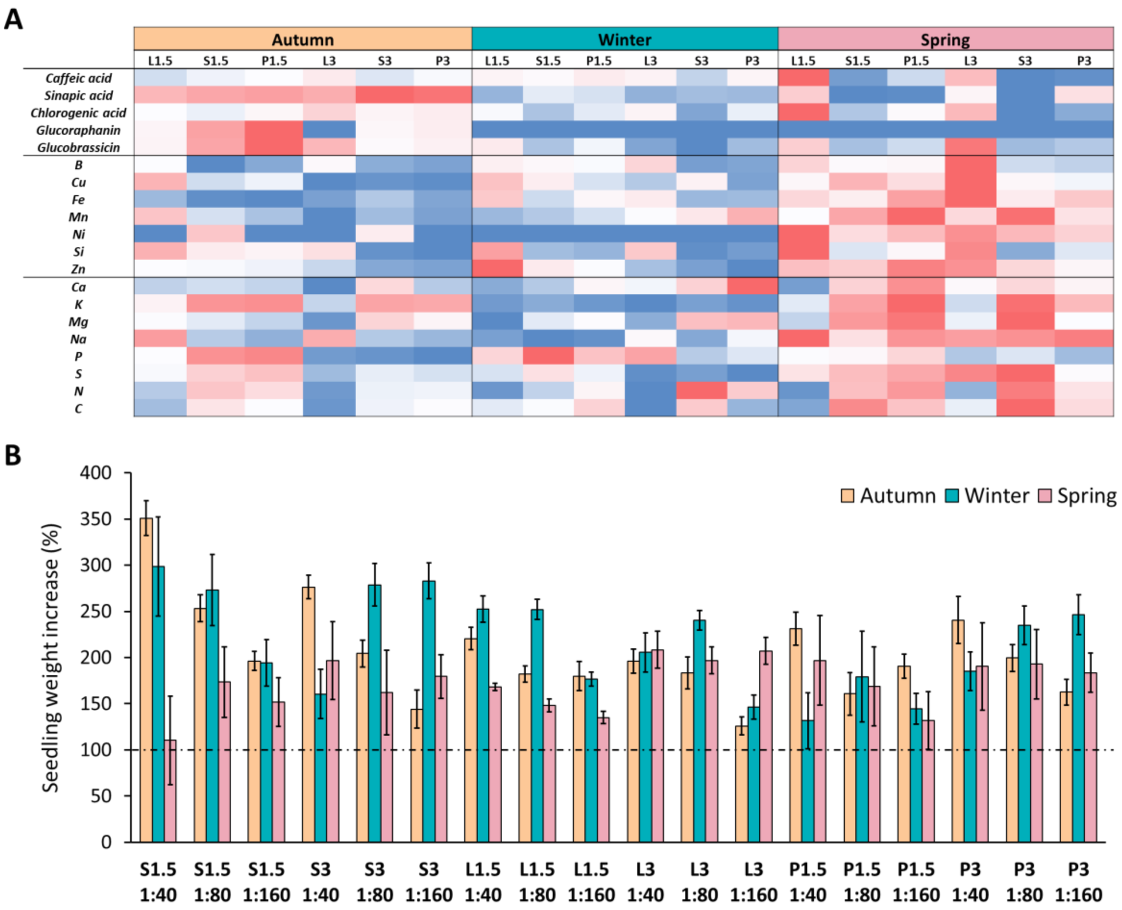

S2 Fig 1. (**A**) Summary of the composition of broccoli extracts obtained from leaves (L), stems (S) and petioles (P) harvested at 1.5 and 3 months in autumn, winter and spring. (**B**) Change (in %) in the biomass of Pak choi seedlings treated with the different extracts compared to untreated seedlings. Data are mean ± SE (n=5-12).
