# Supplementary material for: Valorization of broccoli by-products: seasonal variations in bioactive compounds and their biostimulant effects on pak choi germination
Source: PLoS One. 2025 May 15;20(5):e0323848. doi: 10.1371/journal.pone.0323848 (PMC12101848; doi:10.1371/journal.pone.0323848)
Supplement: S3 Fig — (PDF) [file pone.0323848.s003.pdf]

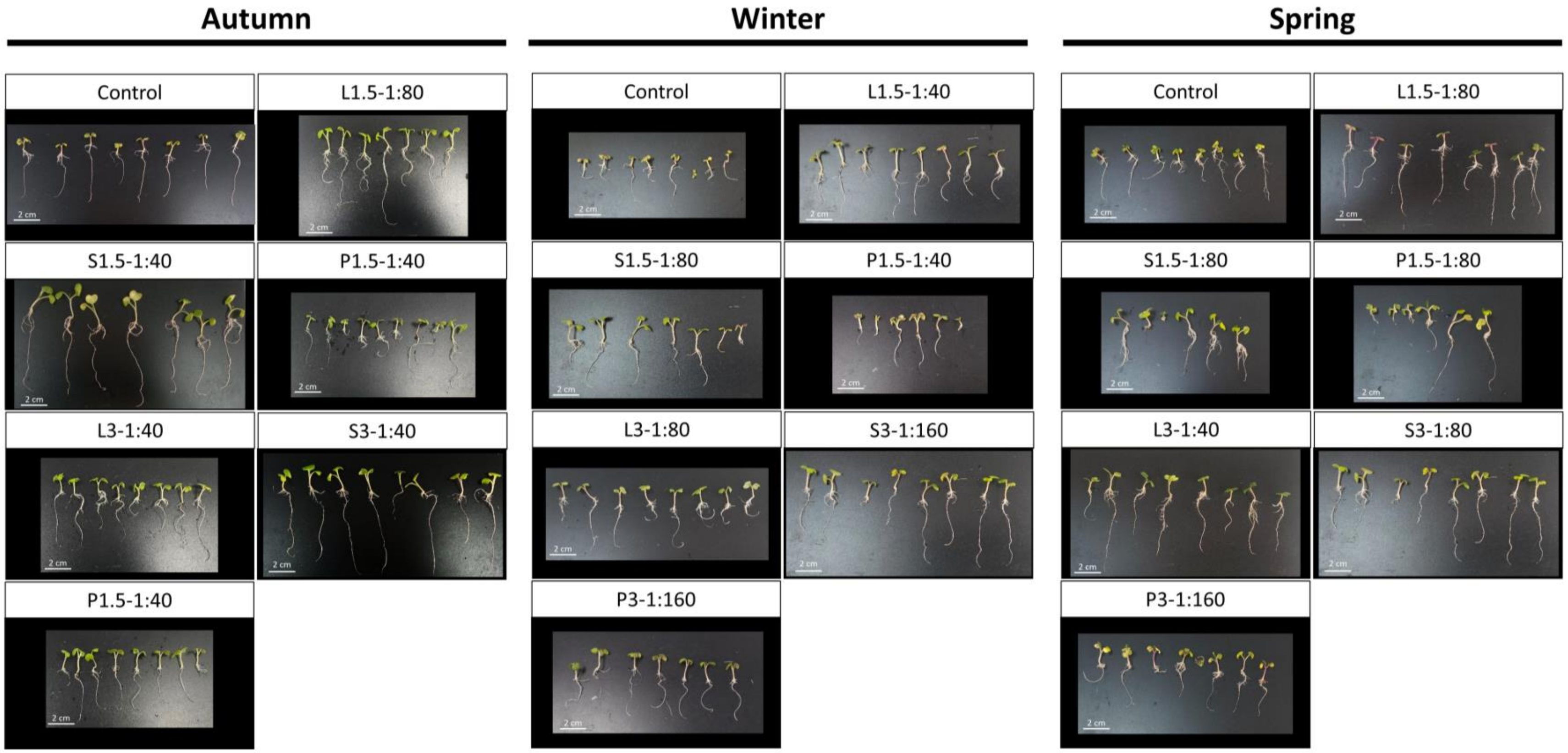

S3 Fig 2. Images of Pak Choi seedlings grown on control agar and on agar with broccoli extracts obtained from leaves (L), stems (S) and petioles (P) harvested at 1.5 and 3 months in autumn, winter and spring.
