# Supplementary material for: Valorization of broccoli by-products: seasonal variations in bioactive compounds and their biostimulant effects on pak choi germination
Source: PLoS One. 2025 May 15;20(5):e0323848. doi: 10.1371/journal.pone.0323848 (PMC12101848; doi:10.1371/journal.pone.0323848)
Supplement: S1 Data — (PDF) [file pone.0323848.s004.pdf]

Temperature and RH			
**Date**	**Hour**	**Temperature (ºC)**	**Relative humity (%)**
30/Oct	09:30	21	39
07/Nov	09:40	27.5	26
14/Nov	09:38	27	39
21/Nov	09:28	23	35
28/Nov	08:24	15.2	53
30/Nov	10:00	20	50
05/Dec	09:29	10.3	55
30/Jan	10:00	16	49
07/Feb	08:15	13.7	44
20/Feb	08:29	12.5	52
29/Feb	09:08	22	28
14/Mar	09:20	27.5	30
20/Mar	08:54	20.7	57
27/Mar	09:43	31.1	21
04/Apr	08:38	19.1	43
11/Apr	9:30	25.6	35
17/Apr	9:22	27.1	40
30/Apr	9:08	23.7	38
08/May	9:30	31.8	25
16/May	8:26	24.5	25
06/Jun	8:10	22.9	47
24/May	8:15	26.8	30

Biomass

FW aerial part (g)

Season	Time (months)	Mean	SD	SE of Mean
Autumn	1.5	246.81818	56.88905	17.15269
Autumn	3	1619.61538	282.06428	78.23055
Winter	1.5	369.375	61.72737	21.82392
Winter	3	1401.66667	213.51229	71.17076
Spring	1.5	537.25	137.01069	48.44059
Spring	3	1018.125	202.99433	71.76933

DW aerial part (g)

Season	Time (months)	Mean	SD	SE of Mean
Autumn	1.5	26.71465	4.84156	2.16521
Autumn	3	165.81038	24.38529	9.95525
Winter	1.5	37.38888	7.95882	3.24917
Winter	3	125.52015	15.71477	7.02786
Spring	1.5	52.01139	26.53773	10.83398
Spring	3	133.55807	49.58055	20.24117

Water content (%)

Season	Time (months)	Mean	SD	SE of Mean
Autumn	1.5	88.97816	2.15874	0.68265
Autumn	3	89.8853	1.47919	0.427
Winter	1.5	89.99892	1.9987	0.66623
Winter	3	90.01542	1.39681	0.49385
Spring	1.5	90.60815	1.70644	0.64497
Spring	3	87.04794	3.10658	1.03553

ICP plants				
**Data**	**A**	**B**	**Mean**	**SE of mean**
B (mg/Kg)	Autumn	1.5	27.64733	1.91688
B (mg/Kg)	Autumn	3	34.50671	1.88127
B (mg/Kg)	Winter	1.5	28.99109	2.26955
B (mg/Kg)	Winter	3	45.23454	4.25149
B (mg/Kg)	Spring	1.5	32.03017	1.19305
B (mg/Kg)	Spring	3	79.79404	2.4983
Cu (mg/Kg)	Autumn	1.5	1.98866	0.07612
Cu (mg/Kg)	Autumn	3	2.47173	0.22207
Cu (mg/Kg)	Winter	1.5	1.91725	0.12982
Cu (mg/Kg)	Winter	3	1.7131	0.13557
Cu (mg/Kg)	Spring	1.5	2.03874	0.25174
Cu (mg/Kg)	Spring	3	2.36095	0.19647
Fe (mg/Kg)	Autumn	1.5	72.72181	4.36478
Fe (mg/Kg)	Autumn	3	72.19084	4.24532
Fe (mg/Kg)	Winter	1.5	65.39013	1.87543
Fe (mg/Kg)	Winter	3	72.36935	3.70626
Fe (mg/Kg)	Spring	1.5	56.54581	5.6839
Fe (mg/Kg)	Spring	3	89.25921	6.23549
Mn (mg/Kg)	Autumn	1.5	120.4394	3.53064
Mn (mg/Kg)	Autumn	3	103.76807	3.5872
Mn (mg/Kg)	Winter	1.5	136.56175	7.98361
Mn (mg/Kg)	Winter	3	145.86479	8.61516
Mn (mg/Kg)	Spring	1.5	119.56061	10.69015
Mn (mg/Kg)	Spring	3	116.04564	11.66566
Ni (mg/Kg)	Autumn	1.5	0.75633	0.16298
Ni (mg/Kg)	Autumn	3	3.37478	0.88338
Ni (mg/Kg)	Winter	1.5	0.24997	0.11717
Ni (mg/Kg)	Winter	3	0.1551	0.01587
Ni (mg/Kg)	Spring	1.5	0.16934	0.03527
Ni (mg/Kg)	Spring	3	0.5516	0.19857
Si (mg/Kg)	Autumn	1.5	211.44802	8.63075
Si (mg/Kg)	Autumn	3	190.93009	15.0167
Si (mg/Kg)	Winter	1.5	344.39786	14.2515
Si (mg/Kg)	Winter	3	420.20701	9.41649
Si (mg/Kg)	Spring	1.5	171.3598	12.28758
Si (mg/Kg)	Spring	3	397.81058	38.01465
Zn (mg/Kg)	Autumn	1.5	27.11859	1.63002
Zn (mg/Kg)	Autumn	3	22.27875	2.02931
Zn (mg/Kg)	Winter	1.5	21.0201	2.21387
Zn (mg/Kg)	Winter	3	20.27338	1.0609
Zn (mg/Kg)	Spring	1.5	23.61148	2.77871
Zn (mg/Kg)	Spring	3	29.51166	2.74461
Ca (g/100g)	Autumn	1.5	2.39356	0.11728
Ca (g/100g)	Autumn	3	2.74141	0.11172
Ca (g/100g)	Winter	1.5	3.29176	0.27735
Ca (g/100g)	Winter	3	4.34697	0.24147
Ca (g/100g)	Spring	1.5	2.18465	0.28104
Ca (g/100g)	Spring	3	2.98568	0.13343

K (g/100g)	Autumn	1.5	4.9844	0.32769
K (g/100g)	Autumn	3	3.30834	0.14011
K (g/100g)	Winter	1.5	4.24906	0.09553
K (g/100g)	Winter	3	3.30599	0.36588
K (g/100g)	Spring	1.5	4.8288	0.4895
K (g/100g)	Spring	3	4.10435	0.21422
Mg (g/100g)	Autumn	1.5	0.53901	0.02895
Mg (g/100g)	Autumn	3	0.45207	0.0159
Mg (g/100g)	Winter	1.5	0.59326	0.03504
Mg (g/100g)	Winter	3	0.54386	0.03267
Mg (g/100g)	Spring	1.5	0.45528	0.05005
Mg (g/100g)	Spring	3	0.60439	0.03497
Na (g/100g)	Autumn	1.5	0.68541	0.03975
Na (g/100g)	Autumn	3	0.8229	0.02535
Na (g/100g)	Winter	1.5	0.75947	0.05721
Na (g/100g)	Winter	3	0.80242	0.03723
Na (g/100g)	Spring	1.5	0.92923	0.12947
Na (g/100g)	Spring	3	0.8791	0.05799
P (g/100g)	Autumn	1.5	0.35766	0.02402
P (g/100g)	Autumn	3	0.32788	0.01915
P (g/100g)	Winter	1.5	0.37479	0.02727
P (g/100g)	Winter	3	0.31875	0.01276
P (g/100g)	Spring	1.5	0.2794	0.02821
P (g/100g)	Spring	3	0.20943	0.02469
S (g/100g)	Autumn	1.5	1.53948	0.04394
S (g/100g)	Autumn	3	1.42132	0.05086
S (g/100g)	Winter	1.5	1.2696	0.01608
S (g/100g)	Winter	3	1.1858	0.05093
S (g/100g)	Spring	1.5	1.34323	0.12605
S (g/100g)	Spring	3	1.3203	0.06843

ICP extract				
**Data**	**A**	**B**	**Mean**	**SE of mean**
B (mg/L)	Autumn	L1.5	2.94798	0.25887
B (mg/L)	Autumn	S1.5	0.98132	0.55653
B (mg/L)	Autumn	P1.5	1.51973	0.31138
B (mg/L)	Autumn	L3	3.40211	0.72476
B (mg/L)	Autumn	S3	1.57773	0.33715
B (mg/L)	Autumn	P3	1.4013	0.36143
B (mg/L)	Winter	L1.5	4.49346	0.10952
B (mg/L)	Winter	S1.5	3.52802	0.45399
B (mg/L)	Winter	P1.5	2.89961	0.00991
B (mg/L)	Winter	L3	7.37169	0.70461
B (mg/L)	Winter	S3	1.33281	0.099
B (mg/L)	Winter	P3	1.44198	0.17479
B (mg/L)	Spring	L1.5	7.63118	0.82005
B (mg/L)	Spring	S1.5	3.29719	0.3052
B (mg/L)	Spring	P1.5	3.60882	0.37256
B (mg/L)	Spring	L3	19.56148	3.96947
B (mg/L)	Spring	S3	2.37258	0.06635
B (mg/L)	Spring	P3	2.20465	0.13151
Cu (mg/L)	Autumn	L1.5	0.13191	0.03152
Cu (mg/L)	Autumn	S1.5	0.06989	0.01995
Cu (mg/L)	Autumn	P1.5	0.08154	0.01379
Cu (mg/L)	Autumn	L3	0.02337	0.00847
Cu (mg/L)	Autumn	S3	0.02894	0.01995
Cu (mg/L)	Autumn	P3	0.0257	0.01622
Cu (mg/L)	Winter	L1.5	0.1232	0.03817
Cu (mg/L)	Winter	S1.5	0.09637	0.00136
Cu (mg/L)	Winter	P1.5	0.07265	0.00163
Cu (mg/L)	Winter	L3	0.05898	0.01545
Cu (mg/L)	Winter	S3	0.09363	0.02379
Cu (mg/L)	Winter	P3	0.03696	0.03696
Cu (mg/L)	Spring	L1.5	0.0909	0.00743
Cu (mg/L)	Spring	S1.5	0.13133	0.01629
Cu (mg/L)	Spring	P1.5	0.10643	0.01057
Cu (mg/L)	Spring	L3	0.17766	0.01788
Cu (mg/L)	Spring	S3	0.09062	0.014
Cu (mg/L)	Spring	P3	0.08299	0.01959
Fe (mg/L)	Autumn	L1.5	0.13171	0.07983
Fe (mg/L)	Autumn	S1.5	0.03634	0.02098
Fe (mg/L)	Autumn	P1.5	0.02372	0.01388
Fe (mg/L)	Autumn	L3	0.07535	0.04511
Fe (mg/L)	Autumn	S3	0.16679	0.14568
Fe (mg/L)	Autumn	P3	0.08696	0.05022
Fe (mg/L)	Winter	L1.5	0.87092	0.07274
Fe (mg/L)	Winter	S1.5	0.20946	0.05012
Fe (mg/L)	Winter	P1.5	0.35897	0.00624
Fe (mg/L)	Winter	L3	0.48348	0.04895
Fe (mg/L)	Winter	S3	0.12942	0.03919
Fe (mg/L)	Winter	P3	0.13576	0.00388

Fe (mg/L)	Spring	L1.5	0.71144	2.92E-04
Fe (mg/L)	Spring	S1.5	0.45381	0.0126
Fe (mg/L)	Spring	P1.5	1.32626	0.12924
Fe (mg/L)	Spring	L3	1.95262	0.0993
Fe (mg/L)	Spring	S3	0.4806	0.02194
Fe (mg/L)	Spring	P3	0.88396	0.22571
Mn (mg/L)	Autumn	L1.5	8.64351	0.91139
Mn (mg/L)	Autumn	S1.5	6.20872	0.33492
Mn (mg/L)	Autumn	P1.5	5.16588	0.54423
Mn (mg/L)	Autumn	L3	3.16239	0.52727
Mn (mg/L)	Autumn	S3	4.92354	1.41257
Mn (mg/L)	Autumn	P3	4.00424	0.94405
Mn (mg/L)	Winter	L1.5	4.79717	0.34473
Mn (mg/L)	Winter	S1.5	5.3699	1.18
Mn (mg/L)	Winter	P1.5	6.20418	0.87518
Mn (mg/L)	Winter	L3	7.16914	0.01007
Mn (mg/L)	Winter	S3	7.73267	0.4742
Mn (mg/L)	Winter	P3	9.15561	0.10341
Mn (mg/L)	Spring	L1.5	7.17501	0.49046
Mn (mg/L)	Spring	S1.5	9.42038	0.58448
Mn (mg/L)	Spring	P1.5	11.00937	0.89061
Mn (mg/L)	Spring	L3	8.09782	1.65319
Mn (mg/L)	Spring	S3	10.83587	0.08238
Mn (mg/L)	Spring	P3	7.8629	1.47928
Ni (mg/L)	Autumn	L1.5	5.00E-13	0
Ni (mg/L)	Autumn	S1.5	0.0225	0.0225
Ni (mg/L)	Autumn	P1.5	5.00E-13	0
Ni (mg/L)	Autumn	L3	5.00E-13	0
Ni (mg/L)	Autumn	S3	0.00697	0.00697
Ni (mg/L)	Autumn	P3	5.00E-13	0
Ni (mg/L)	Winter	L1.5	5.00E-13	0
Ni (mg/L)	Winter	S1.5	5.00E-13	0
Ni (mg/L)	Winter	P1.5	5.00E-13	0
Ni (mg/L)	Winter	L3	5.00E-13	0
Ni (mg/L)	Winter	S3	5.00E-13	0
Ni (mg/L)	Winter	P3	5.00E-13	0
Ni (mg/L)	Spring	L1.5	0.06102	0.01022
Ni (mg/L)	Spring	S1.5	0.01329	0.01329
Ni (mg/L)	Spring	P1.5	0.02727	0.0138
Ni (mg/L)	Spring	L3	0.04504	0.00464
Ni (mg/L)	Spring	S3	0.02827	8.32E-05
Ni (mg/L)	Spring	P3	0.018	0.00902
Si (mg/L)	Autumn	L1.5	34.09022	5.83726
Si (mg/L)	Autumn	S1.5	18.84624	1.32178
Si (mg/L)	Autumn	P1.5	16.82028	2.56736
Si (mg/L)	Autumn	L3	21.8579	5.86447
Si (mg/L)	Autumn	S3	5.61847	1.29799
Si (mg/L)	Autumn	P3	4.95484	1.12852
Si (mg/L)	Winter	L1.5	38.59714	4.98852
Si (mg/L)	Winter	S1.5	9.16933	0.37081

Si (mg/L)	Winter	P1.5	8.44322	0.63613
Si (mg/L)	Winter	L3	27.89863	2.62531
Si (mg/L)	Winter	S3	5.4093	0.48683
Si (mg/L)	Winter	P3	6.49295	0.17271
Si (mg/L)	Spring	L1.5	52.69238	3.80231
Si (mg/L)	Spring	S1.5	12.4811	2.0468
Si (mg/L)	Spring	P1.5	15.99713	2.6859
Si (mg/L)	Spring	L3	44.46137	10.03141
Si (mg/L)	Spring	S3	7.68948	0.62646
Si (mg/L)	Spring	P3	12.66449	3.18622
Zn (mg/L)	Autumn	L1.5	3.57093	0.76068
Zn (mg/L)	Autumn	S1.5	3.49853	0.57152
Zn (mg/L)	Autumn	P1.5	3.35343	0.50677
Zn (mg/L)	Autumn	L3	2.72553	0.76754
Zn (mg/L)	Autumn	S3	1.64469	0.37667
Zn (mg/L)	Autumn	P3	1.49591	0.36196
Zn (mg/L)	Winter	L1.5	6.64951	0.07154
Zn (mg/L)	Winter	S1.5	3.94975	0.62026
Zn (mg/L)	Winter	P1.5	3.55109	0.01224
Zn (mg/L)	Winter	L3	2.19185	0.28446
Zn (mg/L)	Winter	S3	1.46024	0.12279
Zn (mg/L)	Winter	P3	0.9424	0.01194
Zn (mg/L)	Spring	L1.5	4.85059	0.47759
Zn (mg/L)	Spring	S1.5	4.56568	0.37889
Zn (mg/L)	Spring	P1.5	6.1538	0.16356
Zn (mg/L)	Spring	L3	5.81059	1.09557
Zn (mg/L)	Spring	S3	4.35714	0.15412
Zn (mg/L)	Spring	P3	3.70853	0.79104
Ca (mg/L)	Autumn	L1.5	313.01104	30.16795
Ca (mg/L)	Autumn	S1.5	344.04102	74.72586
Ca (mg/L)	Autumn	P1.5	319.89835	55.60256
Ca (mg/L)	Autumn	L3	132.51197	33.50855
Ca (mg/L)	Autumn	S3	548.32377	92.82493
Ca (mg/L)	Autumn	P3	295.83088	55.79748
Ca (mg/L)	Winter	L1.5	217.34698	90.286
Ca (mg/L)	Winter	S1.5	292.80539	86.75324
Ca (mg/L)	Winter	P1.5	447.00744	121.3445
Ca (mg/L)	Winter	L3	401.51686	9.87057
Ca (mg/L)	Winter	S3	574.05764	12.5371
Ca (mg/L)	Winter	P3	947.21829	79.18949
Ca (mg/L)	Spring	L1.5	183.38094	19.85393
Ca (mg/L)	Spring	S1.5	571.32866	78.02697
Ca (mg/L)	Spring	P1.5	808.44405	125.06042
Ca (mg/L)	Spring	L3	436.49382	82.17981
Ca (mg/L)	Spring	S3	554.40333	32.56188
Ca (mg/L)	Spring	P3	451.94698	132.25808
K (mg/L)	Autumn	L1.5	7557.25	1156.29562
K (mg/L)	Autumn	S1.5	14630.75	1294.32239
K (mg/L)	Autumn	P1.5	15025.16667	1607.91507
K (mg/L)	Autumn	L3	5168	700.798

K (mg/L)	Autumn	S3	13442.33333	1259.34945
K (mg/L)	Autumn	P3	13068.5	902.88551
K (mg/L)	Winter	L1.5	2646.6046	204.12351
K (mg/L)	Winter	S1.5	3345.87595	434.6809
K (mg/L)	Winter	P1.5	2521.6532	31.87775
K (mg/L)	Winter	L3	1922.30603	32.11064
K (mg/L)	Winter	S3	2879.7113	70.71674
K (mg/L)	Winter	P3	2304.05053	126.81029
K (mg/L)	Spring	L1.5	6031.86626	456.98415
K (mg/L)	Spring	S1.5	13573.09746	2452.18344
K (mg/L)	Spring	P1.5	17830.0834	1913.88064
K (mg/L)	Spring	L3	5425.47702	912.28285
K (mg/L)	Spring	S3	17366.89822	255.29196
K (mg/L)	Spring	P3	11797.01995	1519.80179
Mg (mg/L)	Autumn	L1.5	853.625	88.61395
Mg (mg/L)	Autumn	S1.5	780	33.30228
Mg (mg/L)	Autumn	P1.5	683	100.38945
Mg (mg/L)	Autumn	L3	415.80769	25.86124
Mg (mg/L)	Autumn	S3	1055.5	135.72245
Mg (mg/L)	Autumn	P3	896.83333	39.83856
Mg (mg/L)	Winter	L1.5	361.08485	23.9252
Mg (mg/L)	Winter	S1.5	779.55722	296.49278
Mg (mg/L)	Winter	P1.5	863.75364	296.24636
Mg (mg/L)	Winter	L3	515.82387	12.20671
Mg (mg/L)	Winter	S3	1155	50
Mg (mg/L)	Winter	P3	1195	20
Mg (mg/L)	Spring	L1.5	683.8927	49.97246
Mg (mg/L)	Spring	S1.5	1344.61489	99.46128
Mg (mg/L)	Spring	P1.5	1508.63536	106.1691
Mg (mg/L)	Spring	L3	808.55615	201.31535
Mg (mg/L)	Spring	S3	1574.9458	29.79996
Mg (mg/L)	Spring	P3	855.65271	120.55256
Na (mg/L)	Autumn	L1.5	1713.375	233.29473
Na (mg/L)	Autumn	S1.5	1025.75	59.65893
Na (mg/L)	Autumn	P1.5	917.5	155.37683
Na (mg/L)	Autumn	L3	1628.125	192.84281
Na (mg/L)	Autumn	S3	1015.875	210.85208
Na (mg/L)	Autumn	P3	945.125	126.27869
Na (mg/L)	Winter	L1.5	849.24773	11.40401
Na (mg/L)	Winter	S1.5	759.61471	77.02997
Na (mg/L)	Winter	P1.5	788.47279	85.42921
Na (mg/L)	Winter	L3	1244.96762	58.65125
Na (mg/L)	Winter	S3	883.25239	20.90514
Na (mg/L)	Winter	P3	1200.59422	47.25063
Na (mg/L)	Spring	L1.5	1972.54693	94.37245
Na (mg/L)	Spring	S1.5	1366.18388	148.6576
Na (mg/L)	Spring	P1.5	1758.97997	151.83934
Na (mg/L)	Spring	L3	1703.11004	219.54255
Na (mg/L)	Spring	S3	1773.36725	107.92951
Na (mg/L)	Spring	P3	1865.82278	189.58046

P (mg/L)	Autumn	L1.5	95.8366	20.7988
P (mg/L)	Autumn	S1.5	133.99692	16.42688
P (mg/L)	Autumn	P1.5	137.41038	13.1894
P (mg/L)	Autumn	L3	43.7814	8.35831
P (mg/L)	Autumn	S3	39.93433	10.83099
P (mg/L)	Autumn	P3	34.46743	12.55928
P (mg/L)	Winter	L1.5	109.63892	4.23113
P (mg/L)	Winter	S1.5	147.86078	7.75951
P (mg/L)	Winter	P1.5	116.3295	4.8104
P (mg/L)	Winter	L3	127.38323	8.15177
P (mg/L)	Winter	S3	69.75122	4.80283
P (mg/L)	Winter	P3	84.04124	16.65085
P (mg/L)	Spring	L1.5	95.51433	11.99721
P (mg/L)	Spring	S1.5	97.60913	11.95074
P (mg/L)	Spring	P1.5	109.4515	8.14578
P (mg/L)	Spring	L3	67.69201	28.91658
P (mg/L)	Spring	S3	83.62214	11.25328
P (mg/L)	Spring	P3	61.63002	6.40648
S (mg/L)	Autumn	L1.5	650.8243	114.32668
S (mg/L)	Autumn	S1.5	758.75	72.44725
S (mg/L)	Autumn	P1.5	794.5	107.91084
S (mg/L)	Autumn	L3	519.83333	25.25921
S (mg/L)	Autumn	S3	626.83333	40.52194
S (mg/L)	Autumn	P3	599.83333	25.83333
S (mg/L)	Winter	L1.5	602.96056	80.20937
S (mg/L)	Winter	S1.5	721.73038	93.89893
S (mg/L)	Winter	P1.5	637.18534	17.9009
S (mg/L)	Winter	L3	427.69159	8.09736
S (mg/L)	Winter	S3	461.97717	0.40179
S (mg/L)	Winter	P3	416.0374	10.42622
S (mg/L)	Spring	L1.5	716.2912	33.68172
S (mg/L)	Spring	S1.5	803.74527	77.0256
S (mg/L)	Spring	P1.5	849.96301	72.29478
S (mg/L)	Spring	L3	927.9897	178.22962
S (mg/L)	Spring	S3	994.53016	58.45861
S (mg/L)	Spring	P3	662.5817	136.52578
Ntotal (g/100g)	Autumn	L1.5	0.3507	0.04243
Ntotal (g/100g)	Autumn	S1.5	0.61451	0.02387
Ntotal (g/100g)	Autumn	P1.5	0.5581	0.09156
Ntotal (g/100g)	Autumn	L3	0.22144	0.03476
Ntotal (g/100g)	Autumn	S3	0.45248	0.10692
Ntotal (g/100g)	Autumn	P3	0.45883	0.07405
Ntotal (g/100g)	Winter	L1.5	0.2134	0.00985
Ntotal (g/100g)	Winter	S1.5	0.3716	0.07145
Ntotal (g/100g)	Winter	P1.5	0.50223	0.03688
Ntotal (g/100g)	Winter	L3	0.17965	0.0345
Ntotal (g/100g)	Winter	S3	0.8356	0.06195
Ntotal (g/100g)	Winter	P3	0.60013	0.07468
Ntotal (g/100g)	Spring	L1.5	0.20885	0.04598
Ntotal (g/100g)	Spring	S1.5	0.62805	0.03829

Ntotal (g/100g)	Spring	P1.5	0.72275	0.04462
Ntotal (g/100g)	Spring	L3	0.28863	0.04728
Ntotal (g/100g)	Spring	S3	0.78835	0.0383
Ntotal (g/100g)	Spring	P3	0.54265	0.05122
Ctotal (g/100g)	Autumn	L1.5	1.63038	0.31486
Ctotal (g/100g)	Autumn	S1.5	2.48083	0.38731
Ctotal (g/100g)	Autumn	P1.5	2.2517	0.46795
Ctotal (g/100g)	Autumn	L3	1.23946	0.09802
Ctotal (g/100g)	Autumn	S3	2.1522	0.46712
Ctotal (g/100g)	Autumn	P3	2.25994	0.33927
Ctotal (g/100g)	Winter	L1.5	2.0722	0.25815
Ctotal (g/100g)	Winter	S1.5	2.23263	0.28323
Ctotal (g/100g)	Winter	P1.5	2.6481	0.00345
Ctotal (g/100g)	Winter	L3	1.11868	0.24988
Ctotal (g/100g)	Winter	S3	2.72873	0.14623
Ctotal (g/100g)	Winter	P3	1.5426	0.1039
Ctotal (g/100g)	Spring	L1.5	1.39545	0.45847
Ctotal (g/100g)	Spring	S1.5	3.34535	0.28201
Ctotal (g/100g)	Spring	P1.5	2.79647	0.17447
Ctotal (g/100g)	Spring	L3	2.11653	0.34074
Ctotal (g/100g)	Spring	S3	3.65743	0.32517
Ctotal (g/100g)	Spring	P3	2.56627	0.33509

Secondary metabolites

Caffeic acid

Season	D	Mean	SE of mean
Autumn	L1.5	0.37002	0.07067
Autumn	S1.5	0.46058	0.0012
Autumn	P1.5	0.51023	0.00787
Autumn	L3	0.81325	0.03002
Autumn	S3	0.40781	0.06684
Autumn	P3	0.49125	0.10789
Winter	L1.5	0.66334	0.04173
Winter	S1.5	0.55695	0.07714
Winter	P1.5	0.79103	0.01615
Winter	L3	0.67456	0.00845
Winter	S3	0.32204	0.03752
Winter	P3	0.64656	0.17291
Spring	L1.5	2.95172	0.19164
Spring	S1.5	0.08354	0.04934
Spring	P1.5	0.35337	0.07367
Spring	L3	1.56954	0.26101
Spring	S3	1.00E-07	0
Spring	P3	0.02952	0.02322

Sinapic acid

Season	D	Mean	SE of mean
Autumn	L1.5	0.39423	0.04631
Autumn	S1.5	0.44488	0.03577
Autumn	P1.5	0.46667	0.0161
Autumn	L3	0.42579	0.02304
Autumn	S3	0.63116	0.05487
Autumn	P3	0.6005	0.01136
Winter	L1.5	0.06811	0.0031
Winter	S1.5	0.15478	0.006
Winter	P1.5	0.1391	0.00373
Winter	L3	0.06287	0.00403
Winter	S3	0.08199	0.0063
Winter	P3	0.07621	0.00804
Spring	L1.5	0.32596	0.04655
Spring	S1.5	1.00E-07	0
Spring	P1.5	0.00554	0.00554
Spring	L3	0.20785	0.10126
Spring	S3	1.00E-07	0
Spring	P3	0.26908	0.15033

Chlorogenic acid

Season	D	Mean	SE of mean
Autumn	L1.5	9.50583	0.93164
Autumn	S1.5	8.78877	0.41055
Autumn	P1.5	10.99895	0.67396
Autumn	L3	18.93119	1.14755
Autumn	S3	11.53683	1.81247

Autumn	P3	13.21266	1.702
Winter	L1.5	9.24583	0.06435
Winter	S1.5	6.4484	0.62647
Winter	P1.5	8.48361	0.13961
Winter	L3	11.47232	0.19231
Winter	S3	4.88711	0.39986
Winter	P3	8.71594	2.14965
Spring	L1.5	43.5403	2.95314
Spring	S1.5	6.61152	0.8066
Spring	P1.5	10.08953	0.95528
Spring	L3	25.11246	1.7249
Spring	S3	3.43999	0.2104
Spring	P3	5.19314	0.25837

Glucoraphanin

Season	D	Mean	SE of mean
Autumn	L1.5	0.09142	0.05278
Autumn	S1.5	1.03104	0.30362
Autumn	P1.5	1.6763	0.09114
Autumn	L3	1.00E-07	0
Autumn	S3	0.07478	0.03752
Autumn	P3	0.1796	0.09876
Winter	L1.5	1.00E-07	0
Winter	S1.5	1.00E-07	0
Winter	P1.5	1.00E-07	0
Winter	L3	1.00E-07	0
Winter	S3	1.00E-07	0
Winter	P3	1.00E-07	0
Spring	L1.5	1.00E-07	0
Spring	S1.5	1.00E-07	0
Spring	P1.5	1.00E-07	0
Spring	L3	1.00E-07	0
Spring	S3	1.00E-07	0
Spring	P3	1.00E-07	0

Glucobrassicin

Season	D	Mean	SE of mean
Autumn	L1.5	15.81705	4.34362
Autumn	S1.5	34.22845	2.4977
Autumn	P1.5	48.41329	1.31063
Autumn	L3	30.07079	4.59131
Autumn	S3	14.33274	0.54446
Autumn	P3	16.60928	0.38592
Winter	L1.5	17.92622	0.51949
Winter	S1.5	7.76556	0.69783
Winter	P1.5	12.90348	0.3834
Winter	L3	4.63113	0.42642
Winter	S3	1.75968	0.24947
Winter	P3	7.02312	5.18597
Spring	L1.5	24.62299	2.20648

Spring	S1.5	7.40162	0.86105
Spring	P1.5	10.20842	0.69275
Spring	L3	44.95152	4.71969
Spring	S3	6.8991	0.45134
Spring	P3	8.29899	3.98364

Germination assays

Autumn

Sample	Dilution	Mean	SE of mean
C1	0	30.975	1.92917
C2	0	30.975	1.92917
C3	0	30.975	1.92917
L1.5	1:40	68.34286	3.71297
L1.5	1:80	56.45	2.7627
L1.5	1:160	55.75	4.80472
L3	1:40	60.6875	4.0354
L3	1:80	56.7875	5.29111
L3	1:160	39	3.03509
S1.5	1:40	108.725	5.79691
S1.5	1:80	78.41429	4.50764
S1.5	1:160	56.475	5.19649
S3	1:40	85.6125	3.88272
S3	1:80	63.325	4.53115
S3	1:160	44.675	6.42708
P1.5	1:40	71.675	5.4931
P1.5	1:80	49.75	7.14738
P1.5	1:160	59.05	3.95402
P3	1:40	67.12857	9.91321
P3	1:80	57.4375	5.81451
P3	1:160	50.3375	4.31016

Winter

Sample	Dilution	Mean	SE of mean
C1	0	21.24375	1.7187
C2	0	21.24375	1.7187
C3	0	21.24375	1.7187
L1.5	1:40	53.65	2.99619
L1.5	1:80	53.5625	2.28488
L1.5	1:160	37.55	1.65097
L3	1:40	50.95	4.55132
L3	1:80	51.0375	2.25293
L3	1:160	28.975	3.17725
S1.5	1:40	73.85	5.9139
S1.5	1:80	58	8.19971
S1.5	1:160	44.8	4.6091
S3	1:40	34.08333	5.67476
S3	1:80	59.21667	4.91361
S3	1:160	60.125	4.14288
P1.5	1:40	27.9375	6.46438
P1.5	1:80	38.1	10.48296
P1.5	1:160	30.6625	3.53806
P3	1:40	39.3625	4.44265
P3	1:80	49.925	4.45665
P3	1:160	52.3	4.57224

Spring

Sample	Dilution	Mean	SE of mean
C1	0	31.10667	1.34499
C2	0	31.10667	1.34499
C3	0	31.10667	1.34499
L1.5	1:40	51.9875	1.19455
L1.5	1:80	45.7625	2.18542
L1.5	1:160	41.7625	2.12325
L3	1:40	57.56667	5.52761
L3	1:80	66.23333	5.09136
L3	1:160	64.0625	4.44442
S1.5	1:40	34.15	14.80353
S1.5	1:80	53.6125	11.77396
S1.5	1:160	46.9	8.15347
S3	1:40	60.8	13.04291
S3	1:80	50.15	14.11797
S3	1:160	55.5	7.30835
P1.5	1:40	60.8375	14.99875
P1.5	1:80	52.175	13.25941
P1.5	1:160	40.7375	9.69405
P3	1:40	58.8875	14.67205
P3	1:80	59.6625	11.63555
P3	1:160	56.775	6.57598
